# Sensitive and Simplified Detection of Antibiotic Influence on the Dynamic and Versatile Changes of Fecal Short-Chain Fatty Acids

**DOI:** 10.1371/journal.pone.0167032

**Published:** 2016-12-01

**Authors:** Xiaoya Zhao, Zhenzuo Jiang, Fan Yang, Yan Wang, Xiumei Gao, Yuefei Wang, Xin Chai, Guixiang Pan, Yan Zhu

**Affiliations:** 1 Tianjin State Key Laboratory of Modern Chinese Medicine, Tianjin University of Traditional Chinese Medicine, Tianjin, China; 2 Research and Development Center of TCM, Tianjin International Joint Academy of Biotechnology and Medicine, Tianjin, China; Universite Paris-Sud, FRANCE

## Abstract

Short-chain fatty acids (SCFAs), produced by anaerobic fermentation of mainly indigestible dietary carbohydrates by gut microbiota, have a profound influence on intestinal function and host energy metabolism. Antibiotics may seriously disturb the balance of fecal SCFAs. To evaluate the impacts of antibiotics on fecal SCFAs produced by gut microbiota, a simple, reproducible and accurate gas chromatography (GC) method, which can simultaneously analyze seven SCFAs in fecal samples, was developed and validated. The ranges of detection and quantitation of the SCFAs reached 0.0868 ~ 0.393 and 0.261 ~ 1.18 μg·mL^-1^ respectively, in an optimized protocol for SCFAs extraction and analysis that used 10 mL 75% ethanol aqueous solution containing 1% HCl, without ultrasonication. The technique exhibited excellent intra-day (relative standard deviation (RSD) ≤ 2.54%) and inter-day (RSD ≤ 4.33%) precisions for all the SCFAs. Later, we administered broad-spectrum antibiotics, cefdinir or azithromycin to rats and analyzed the alterations in fecal SCFAs. The total amount, types and distribution of nearly all fecal SCFAs were significantly altered during the administration and even after withdrawal of the antibiotics in rats. The effects of cefdinir on the SCFAs were more pronounced than those of azithromycin. Our findings suggest SCFAs may serve as sensitive indicators to monitor the influences of antibiotics on SCFAs originated by intestinal bacteria. Our improved SCFAs analysis method is a potential platform for a standard clinical test of the effects of new antibiotics on SCFAs.

## Introduction

Gut microbiota, a complex microbial community densely inhabits in the intestinal tract, plays essential roles in our health via multiple mechanisms, one of which is to provide functional metabolites for the host. Short-chain fatty acids (SCFAs) are major end-products of anaerobic fermentation by microbiota in the large intestine, including acetic acid, propionic acid, isobutyric acid, butyric acid, isovaleric acid, valeric acid and hexanoic acid [1–3]. Acetic acid is produced by most enteric bacteria, such as acetogenic bacteria through the Wood-Ljungdahl pathway [4]. But propionic acid is mainly produced by *Bacteroides* and *Propionibacterium* through succinate pathway [5]. Butyrate-producing bacteria mainly include *Faecalibacterium*, *Eubacterium*, and *Roseburia* genera [6], whereas *Roseburia Inulinivorans* and *Coprococcus Catus* are able to produce propionic acid and butyric acid [7]. A recent study showed that most dominant species of the *Bacteroides* positively associated with fecal concentrations of isovaleric and isobutyric acid, which are negatively correlated with blood levels of triglycerides [8]. SCFAs are important anions in the colonic lumen not only to provide the major source of energy for colonocytes [9], but also to exert anti-inflammatory [10] effects, which influence various functions of the intestinal tract [11–13]. In addition, some SCFAs could regulate the growth of known pathogens [14]. Therefore, SCFAs may reflect the metabolic activity of intestinal microbiota [15], which could be modified by dietary and environmental changes [16], particularly by antibiotics [17] and diet [18,19]. Recent studies indicate that SCFAs are produced by gut microbial fermentation of dietary fiber [20], thus, compared to the plant-based diet, an animal-based diet resulted in significantly lower levels of the products of carbohydrate fermentation [18].

Antibiotics plays an effective role in controlling infections caused by pathogens. However, antibiotics also cause adverse effects, including the emergence of resistant bacteria [21] and dysbiosis [22–27]. For example, Young and Schmidt [25], who investigated change of bacterial populations in fecal samples from patients with antibiotic-associated diarrhea by amoxicillin-clavulanic acid treatment, found a reduction of genus *Faecalibacterium* and absence of *Clostridium* on the fourth day of antibiotic treatment. Small sub-unit rRNA sequencing is often employed to determine the community structure of the gut microbiota to monitor microbial community and diversity after antibiotic administration [23,28]. Antibiotics may therefore also affect the intestinal presence and balance of the major metabolites of gut microbiota, including SCFAs, vitamins [29], and amino acids [30]. Previous studies have used a number of techniques for SCFAs analysis, including gas chromatography (GC) [31,32], gas chromatography-mass spectrometry (GC-MS) [33], high performance liquid chromatography (HPLC) [34], liquid chromatography–mass spectrometry (LC-MS) [35], capillary electrophoresis (CE) [36], and stable isotope tracer methods [37]. GC is the most common technique to analyze volatile compounds, but it is usual to pretreat feces by derivatization with different reagents before analysis [33,38]. Derivatization is time-consuming and affects the accuracy and reproducibility of the assay. Although several methods have been developed to improve SCFAs determination, a more efficient and simplified approach is still in demand.

Here, we aimed to develop a simple, accurate and robust GC method for the analysis of fecal SCFAs, and to investigate the effects of antibiotics on the fecal SCFAs of rats. Two representative broad-spectrum antibiotics were selected: cefdinir, an advanced-generation cephalosporin, and azithromycin, a macrolide. We found all the SCFAs decreased upon cefdinir or azithromycin treatment and most of the SCFAs did not recover to control levels within 8 days after withdrawal of the antibiotics. The changes of fecal SCFAs may be used as a clinical index to guide antibiotic use.

## Materials and Methods

### Chemicals and reagents

Acetic acid, propionic acid, isobutyric acid, butyric acid, isovaleric acid, valeric acid, hexanoic acid, 2-ethyl butyric acid and 2-ethyl hexanoic acid were purchased from Sigma-Aldrich (St. Louis, MO, USA). We used 2-ethyl butyric acid and 2-ethyl hexanoic acid as internal standards (IS) for final determination of the quantity of each compound present in fecal samples. Ethanol was purchased from Tianjin Damao Chemical Reagent Factory (Tianjin, China). Hydrochloric acid was from Tianjin Jindong Tianzheng Precision Chemical Reagent Factory (Tianjin, China). Ultra-pure water was generated using a Milli-Q water purification system (Millipore, USA). Antibiotics employed were cefdinir dispersible tablets (Shenzhen Zhijun Pharmaceutical Co., Ltd., production batch number A150103), and azithromycin dispersible tablets (Harbin Pharmaceutical Group, Sanchine Nuojie Pharmaceutical Co., Ltd., production batch number 1502224), which were provided by first teaching hospital of Tianjin university of TCM (Tianjin, China).

### Animals

Eighteen male Sprague-Dawley rats weighing 190 ~ 210 g were supplied by Vital River Lab Animal Co., Ltd. (Beijing, China) [Certification number: SCXK (Jing) 2012–0001]. Rats were housed individually in metabolic cages in conditions of controlled temperature (24 ± 1°C), humidity (50−60%) and photoperiod (12 hours light/dark) with free access to drinking water and food pellets (standard chow: 18.0% protein, 4.0% lipids, and 5.0% fiber) during the experiments.

### Experimental design

The 18 rats were randomly divided into three groups, two experimental groups and a control group. Dispersible tablets of cefdinir (135 mg·kg^-1^·d^-1^) and azithromycin (450 mg·kg^-1^·d^-1^) dissolved in saline were respectively gavaged to the two experimental groups of rats every day for 6 days. The control group was treated by gavage with an equivalent volume of saline. In the next 8 days after withdrawal of the antibiotics, the rats accessed water and food freely under the conditions described above. Fecal samples were collected in the following 24h after the first administration, which is labeled as day 1. Subsequently, the rest feces were collected every 2 days at 9 a.m. and labeled as days 3, 5, 7, 9, 11 and 13. Fecal samples were respectively transferred into 4 mL eppendorf tubes and immediately stored at −80°C. Rats were weighed at 9 a.m. during the period of drug administration and recovery.

### Standard solutions

Individual stock standard solutions of acetic acid, propionic acid, isobutyric acid, butyric acid, isovaleric acid, valeric acid, hexanoic acid, 2-ethyl butyric acid and 2-ethyl hexanoic acid were prepared by dissolving the analytes in 75% ethanol aqueous solution, the concentration of which were 23.58, 5.34, 0.46, 9.96, 0.70, 1.28, 1.04, 1.43, and 0.19 mg·mL^-1^. 2-ethyl butyric acid was employed as internal standard for determination of acetic acid, propionic acid and butyric acid. 2-ethyl hexanoic acid was chosen as internal standard for quantitation of isobutyric acid, isovaleric acid, valeric acid and hexanoic acid.

Working standard solutions were prepared and serially diluted with 1% HCl/75% ethanol aqueous solution containing 2-ethyl butyric acid and 2-ethyl hexanoic acid at a final concentration of 44.51 and 2.26 μg·mL^-1^, respectively. The detail concentrations of 7 analytes were as following: acetic acid (33.16 ~ 1061 μg·mL^-1^), propionic acid (7.51 ~ 240.3 μg·mL^-1^), isobutyric acid (0.78 ~ 25.08 μg·mL^-1^), butyric acid (9.34 ~ 298.7 μg·mL^-1^), isovaleric acid (0.98 ~ 31.37 μg·mL^-1^), valeric acid (1.61 ~ 51.36 μg·mL^-1^), and hexanoic acid (0.98 ~ 31.26 μg·mL^-1^), respectively. Once prepared, the solutions were stored at −20°C until analysis.

### Sample solution preparation

As follows, the sample preparation procedure was optimized in terms of extraction solvent, extraction volume, the percentage concentration of HCl in sample solution, and ultrasonication time.

Ultra-pure water (2.5 mL) was added to each 0.5 g fecal sample in a 15 mL tube, vortexed briefly, and then a total of 7.5 mL 1.33% HCl/ethanol solution containing internal standards were added to give the final concentration of 44.51 μg·mL^-1^ (2-ethyl butyric acid) and 2.26 μg·mL^-1^ (2-ethyl hexanoic acid). The samples were homogenized for 2 min, and centrifuged at 17,968 × *g* for 10 min at ambient temperature. Supernatant was promptly transferred to a 2 mL sample vial.

### GC analysis

Analysis was performed using a GC-2010 (Shimadzu, Kyoto, Japan) system equipped with an AOC-20i autosampler, and coupled to flame ionization detector. The chromatographic separation was performed on a DB-FFAP capillary column (30 m × 0.25 mm × 0.25 μm; Agilent Technologies, Palo Alto, CA, USA). A glass liner stoppered with a glass wool plug in the middle of the liner was used to prevent contamination of the GC column with nonvolatile fecal materials. Three microliters of the sample solution was injected in split mode at a ratio of 50:1. The injection temperature and detector temperature were 250°C. The initial temperature of the column was 50°C for 1 min, increased to 120°C at a rate of 15°C·min^-1^, to 170°C at a rate of 5°C·min^-1^, and then to 240°C at a rate of 15°C·min^-1^ for 3 min. The total run time was 23.33 min. The carrier gas was N_2_ (99.999%) at 1 mL·min^-1^. The make-up gas (N_2_) was at 30 mL·min^-1^. The flow rate of H_2_ and air were 47 mL·min^-1^ and 400 mL·min^-1^, respectively.

### Method validation

The method established in this study was validated with method of internal standard for linearity, precision (intra- and inter-day), stability, repeatability and recovery yield, except for limit of detection (LOD) and limit of quantification (LOQ). Calibration curves were constructed based on the peak area ratios of the analytes to the IS (*y*) versus the corresponding concentrations (*x*) of seven standard solutions at different concentrations. The LOD and LOQ were determined at a signal-to-noise ratio (S/N) of about 3 and 10, respectively. The intra- and inter-day precisions of the sample solution were conducted with six replicate injections performed on the same day and on three consecutive days, respectively. The stability of the sample solution stored in the GC autosampler at room temperature was investigated by replicate injection at 0, 2, 4, 6, 8, 10 and 12 h. To confirm the repeatability, six replicates of the same sample were processed and analyzed. A recovery test was used to further evaluate the accuracy of the method.

### Data processing

Area under concentration *versus* time curve was calculated by DAS 3.0 software (Data Analysis System). Line charts and histograms were analyzed with GraphPad Prism 5.01 (GraphPad Software, Inc., USA) and pie graphs were performed by Microsoft Excel 2010 (Microsoft Corporation, USA). Nonparametric *t*-test was used on every antibiotic group and control group comparison. Statistical significance was accepted at *p* < 0.05.

### Ethical statement

This article does not contain any studies with human participants performed by any of the authors. All procedures performed in studies involving animals were in accordance with the ethical standards of Tianjin International Joint Academy of Biotechnology and Medicine (TJAB-TJU20140006).

## Results and Discussion

### Optimization of sample preparation

SCFAs, especially acetic, propionic and butyric acid, are vital to maintain the normal function of intestine and human body. Acetate is absorbed and transported to the liver and peripheral tissues, less metabolized in the colon and acts as substrate for cholesterol synthesis and lipogenesis [39]. Propionate is a primary precursor for gluconeogenesis and it reduces the synthesis of hepatic cholesterol [40]. Butyric acid is a preferred energy source for colonocytes and protects from inflammation [41]. Most of butyrate is metabolized by the colonocyte. Butyrate also affects the regulation of cellular proliferation and apoptosis, resulting in reduced risk of colon cancer [42]. Therefore, we focused on these interesting SCFAs in this study.

The single variable method was used to successively inspect the effect on extraction efficiency of SCFAs from the feces of rats from four sample preparation factors, including (1) the ratio of ethanol to water (25%:75%, 50%:50%, 75%:25% and 100%:0%; v/v); (2) the percentage of HCl (0.1%, 0.5%, 1% and 1.5%); (3) ultrasonication time (0, 5, 10 and 15 min); and (4) extraction volume (5, 10, 20 and 40 mL). Duplicate samples were prepared for each condition to ensure the repeatability of the method.

**Fig 1:**
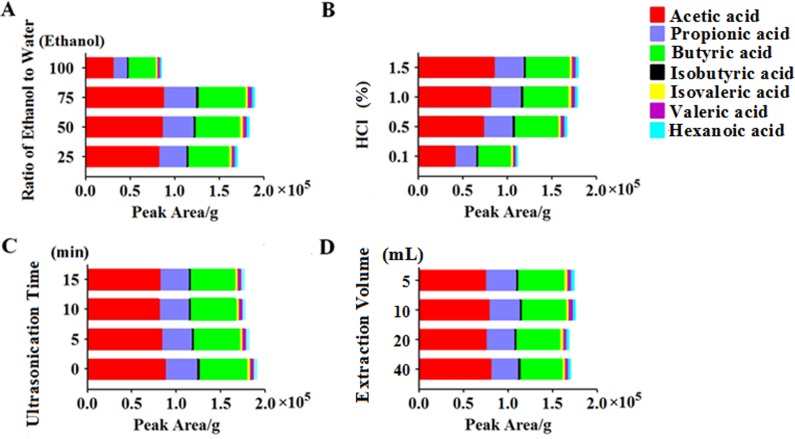
Optimization of the sample extraction method. (A) ratio of ethanol to water; (B) the percentage of HCl; (C) ultrasonication time; and (D) extraction volume.

The ratio of ethanol to water (Fig 1A) and the percentage of HCl (Fig 1B) both had obvious influences on the extraction results. The ultrasonication time (Fig 1C) and the extraction volume (Fig 1D) had no significant effect, but when the samples were diluted with an extraction volume of 10 mL, without ultrasonication, the detection level of SCFAs was increased by 4.3% and 8.2%, respectively. The optimum conditions for extracting SCFAs from rat feces were: extraction in 10 mL 75% ethanol aqueous solution containing 1% HCl, without ultrasonication.

Previous studies reported that fecal SCFAs could be extracted by one-step or more step derivatization [31–38]. The development of reported methods was an essential issue for investigation of SCFAs in different biological samples, which provided more options for analysts. Nonetheless, these methods suffered from limits of the complicated and time-consuming procedure for derivatization [33, 38], or long analytical time [33, 35, 38], or unsuitability for analysis of fat soluble SCFAs [32], or poor sensitivity by UV detection [36], or focus on the determination of SCFAs in other biological samples beyond feces [31, 34–37]. Thus, we developed a simple, rapid and more suitable method for accurate quantification for SCFAs in feces.

### Method validation

**Fig 2:**
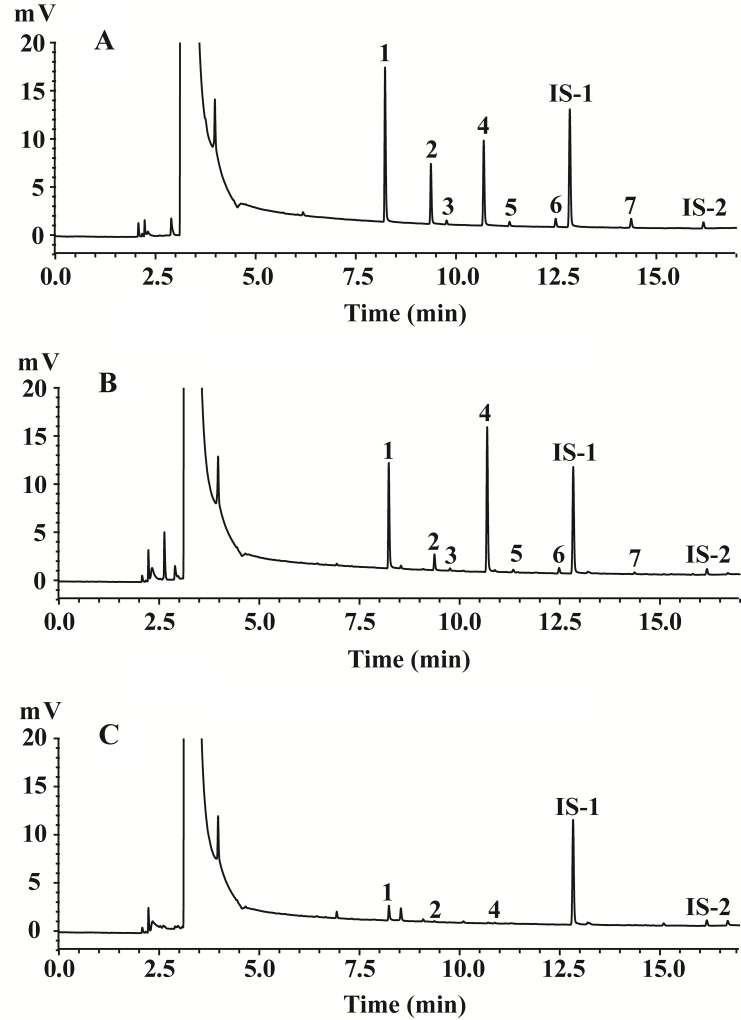
Representative gas chromatograms of samples. (A) mixed standards solution; (B) the control fecal sample; (C) the fecal sample from the cefdinir-treated group on the first day. 1. acetic acid; 2. propionic acid; 3. isobutyric acid; 4. butyric acid; 5. isovaleric acid; 6. valeric acid; 7. hexanoic acid; IS-1. 2-ethyl butyric acid; IS-2. 2-ethyl hexanoic acid.

Using the optimized sample extraction and GC methods, we acquired chromatograms for the mixed standards solution (Fig 2A), the control fecal sample (Fig 2B) and the cefdinir-treated sample (Fig 2C) on day 1. A correlation coefficient (*r*) >0.999 was obtained for all the calibration curves (Table 1). LOD and LOQ of all the SCFAs were 0.0868 ~ 0.393 and 0.261 ~ 1.18 μg·mL^-1^ respectively, indicating that the method is sufficiently sensitive for analyzing fecal SCFAs. The new method exhibited excellent precision (intra-day and inter-day RSD ≤2.54% and ≤4.33%, respectively) (Table 2). The result showed that the SCFAs were stable over 12 h at room temperature (Table 2), and showed an excellent repeatability with RSD ≤4.40% (Table 2). The average recovery values for each SCFAs varied from 92.81 to 104.1% (*n* = 6) with RSD ≤3.98% (Table 2).

**Table 1:** Linearity, LOD and LOQ of seven SCFAs

Compounds	Calibration curves^a^	Linear range	*r*^2^ ^b^	LOQ	LOD
		(μg·mL^-1^)		(μg·mL^-1^)	(μg·mL^-1^)
**Acetic acid**	*y* = 0.0080*x* + 0.1646	33.16 ~ 1061	0.9990	1.18	0.393
**Propionic acid**	*y* = 0.0130*x* + 0.0399	7.51 ~ 240.3	0.9992	0.801	0.267
**Isobutyric acid**	*y* = 0.3367*x -* 0.0810	0.78 ~ 25.08	0.9999	0.684	0.228
**Butyric acid**	*y* = 0.0176*x* + 0.0067	9.34 ~ 298.7	0.9999	0.498	0.166
**Isovaleric acid**	*y* = 0.3997*x -* 0.1440	0.98 ~ 31.37	0.9998	0.523	0.174
**Valeric acid**	*y* = 0.3392*x -* 0.0841	1.61 ~ 51.36	0.9999	0.963	0.321
**Hexanoic acid**	*y* = 0.3907*x -* 0.1201	0.98 ~ 31.26	0.9995	0.261	0.0868

a: *x* concentration (μg·mL^-1^); *y* peak area ratio (area of each SCFA/area of IS)

b: Linearity was expressed as the square of correlation coefficient between concentration and peak area (area of each SCFA/area of IS) counts.

**Table 2:** Intra- and inter-day precision, stability, repeatability and recovery of seven SCFAs

Compounds	Precision, RSD (%)		Stability,	Repeatability,	Recovery (*n* = 6)	
	Intra-day,	Inter-day,	RSD (%),	RSD (%),	Recovery (%)	RSD (%)
	(*n* = 6)	(*n* = 3)	(*n* = 7)	(*n* = 6)		
**Acetic acid**	1.63	3.03	6.37	3.20	92.81	3.98
**Propionic acid**	1.07	3.76	5.47	3.95	94.50	1.78
**Isobutyric acid**	2.07	3.79	1.89	2.39	100.5	3.63
**Butyric acid**	0.70	3.28	3.17	3.10	97.36	1.38
**Isovaleric acid**	2.54	3.93	1.64	3.01	104.1	2.96
**Valeric acid**	1.25	4.33	2.85	4.40	101.5	2.96
**Hexanoic acid**	1.03	2.84	1.70	2.80	98.37	1.49

### The variation in the weight of the rats

**Fig 3:**
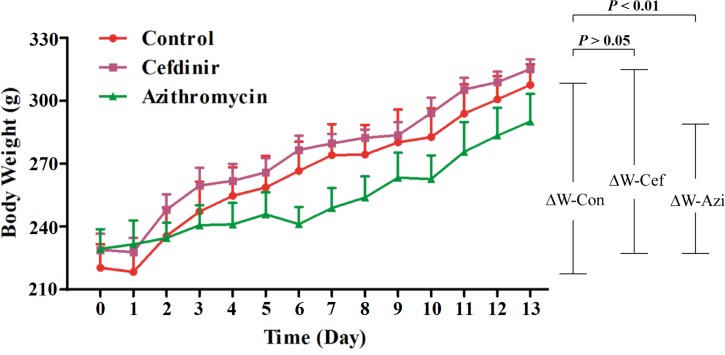
Bodyweight variation in the control, cefdinir- or azithromycin-treated groups. ΔW-Con: weight gain in the control groups. ΔW-Cef: weight gain in the cefdinir-treated groups. ΔW-Azi: weight gain in the azithromycin-treated groups. All measurements were between day 0 and day 13.

The variation in the weight of rats of the control, cefdinir and azithromycin treatment groups was shown in Fig 3. There were significant increases in body weight for all groups of rats from Day 0 to Day 13. During this experimental period, the weight gain of rats was no significantly different between control and cefdinir-treated group (*p* >0.05), but was significantly different between control and azithromycin-treated group (*p* <0.01). Studies have shown that azithromycin cause gastrointestinal side effects [43], but few study report adverse effects of cefdinir related to loss of appetite. Our observation of decreased weight gain in azithromycin-treated rats in this study is consistent with its reported gastrointestinal side effects [43].

### Antibiotic treatments alter fecal SCFAs

The validated GC method was subsequently applied to analyze fecal SCFAs over time. The feces of 18 rats were collected for three days prior to the administration of antibiotics to carry out the analysis of seven SCFAs. As normal level of SCFAs, the average concentrations were 2768.6 ± 106 μg·g^-1^ for acetic acid, 758.1 ± 34.4 μg·g^-1^ for propionic acid, 43.58 ± 0.89 μg·g^-1^ for isobutyric acid, 951.1 ± 32.3 μg·g^-1^ for butyric acid, 53.03 ± 1.22 μg·g^-1^ for isovaleric acid, 75.49 ± 3.18 μg·g^-1^ for valeric acid, and 62.00 ± 1.52 μg·g^-1^ for hexanoic acid. The detail results of fecal SCFAs of rats at day 1, 3, 5, 7, 9, 11, 13 during the period of drug administration and post-drug administration were displayed in Tables A-G in S1 File. The data of seven SCFAs was summarized in Table 3. Treated with cefdinir and azithromycin, the pronounced changes of SCFAs were detected compared with the control group. These variations were found to be in line with other studies performed with other antibiotics, such as ampicillin and clindamycin [15].

**Table 3:** Concentrations of SCFAs in feces of rats from control, cefdinir and azithromycin groups (Conc. Min ~ Max; μg·g^-1^)

Group	Acetic	Propionic	Isobutyric	Butyric	Isovaleric	Valeric	Hexanoic
	acid	acid	acid	acid	acid	acid	acid
**Control**	1354.8 ~ 3810.8	216.6 ~ 1960	8.60 ~ 68.7	241.3 ~ 2562	9.23 ~ 130	12.83 ~ 120.9	8.917 ~ 23.27
**Cefdinir**	287.99 ~ 2888.5	41.23 ~ 1402	8.44 ~ 54.2	68.43 ~ 1014	14.2 ~ 67.2,	9.618 ~ 69.11	8.826 ~ 10.42
**Azithromycin**	282.66 ~ 2467.2	46.05 ~ 965.9	7.25 ~ 27.0	70.96 ~ 1257	8.25 ~ 48.9	9.280 ~ 21.06	9.993 ~ 13.39

**Fig 4:**
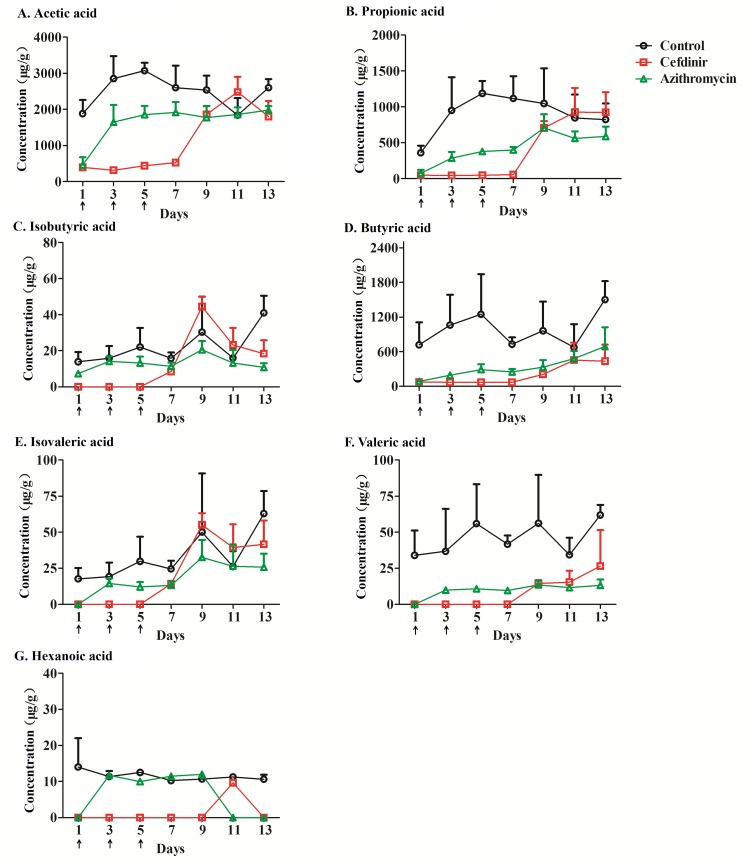
The concentrations of seven SCFAs extracted from fecal samples as a function of time. **Antibiotics (cefdinir or azithromycin) were administered for the first 6 days.** (A) acetic acid; (B) propionic acid; (C) isobutyric acid; (D) butyric acid; (E) isovaleric acid; (F) valeric acid; and (G) hexanoic acid. Arrow means administration of antibiotic.

Fig 4 shows time courses of the changes in the seven SCFAs levels in fecal samples from control rats and rats treated with cefdinir or azithromycin. During cefdinir administration, the concentrations of all the SCFAs were remarkably decreased relative to the controls. Acetic acid, propionic acid and butyric acid were detected in much lower level than those from control rats, while isobutyric acid, isovaleric acid, valeric acid and hexanoic acid were undetectable, suggesting a pronounced variation in the gut microflora of the rats. After cefdinir administration ended, the concentrations of acetic acid, propionic acid, isobutyric acid, butyric acid, isovaleric acid and valeric acid gradually recovered compared with those in the control group, but the levels of butyric acid and valeric acid remained lower than those in the control group. On azithromycin treatment, the concentrations of all SCFAs (except hexanoic acid) declined to below the control levels during the experiment. The fecal SCFAs concentrations during the recovery period (post-drug administration) were higher than those during the period of administration, but still lower than the respective concentrations in the control group, suggesting that the gut microbiota recovered, but did not reach their normal level within 8 days following withdrawal of azithromycin.

Few studies systematically explored the influences of antibiotics on fecal SCFAs [44], and none on the influences of cefdinir or azithromycin. A very recent report showed that the microbiota of preterm infant involves a concomitant alteration in the levels of SCFAs [45]. Moreover, it can be further affected by perinatal antibiotic use, for example, a preterm infants’ mother who received penicillin, ampicillin, or ampicillin plus erythromycin [17]. Our results showed that nearly all the SCFAs were impacted by cefdinir or azithromycin, the two antibiotics widely used in clinics. Our data also indicate that SCFAs could be a sensitive indicator of the influences of antibiotics on gut microbiota.

### Variations of SCFAs by area under concentration *versus* time curves (AUC)

**Fig 5:**
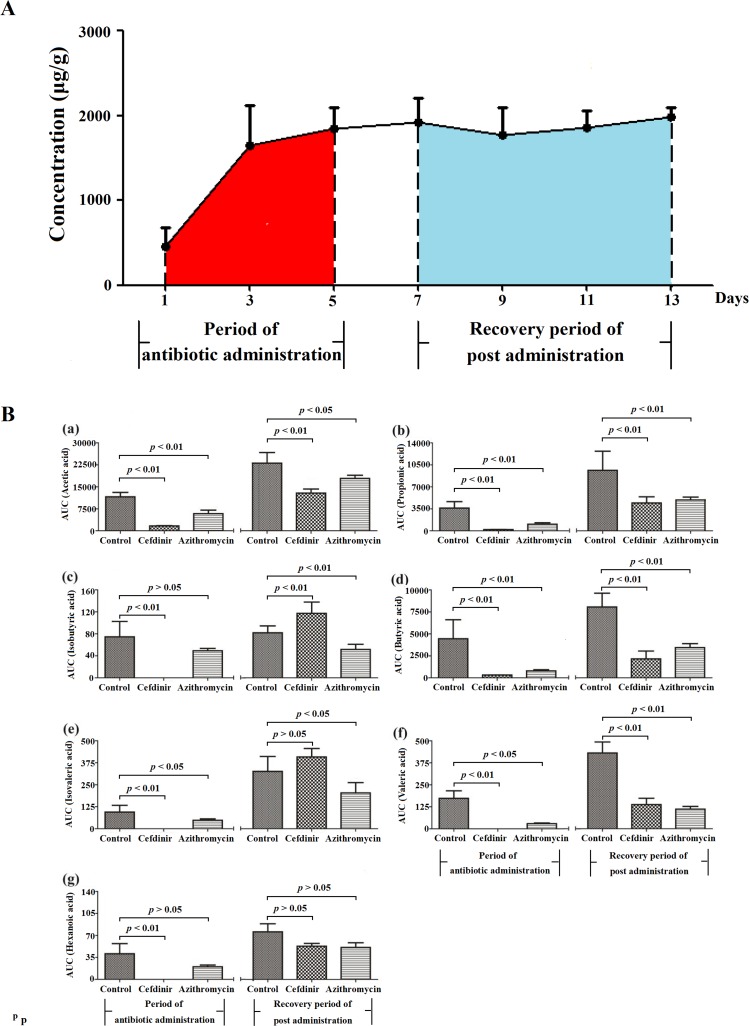
**The schematic diagram of AUC with a function of time (A), and AUC of SCFAs in fecal samples in the periods of antibiotic-treated and recovery (B).** (a) acetic acid; (b) propionic acid; (c) isobutyric acid; (d) butyric acid; (e) isovaleric acid; (f) valeric acid; (g) hexanoic acid.

To further clarify the impact of antibiotics on SCFAs, an area under concentration *versus* time curve (AUC, μg·g^-1^·d) of SCFAs is introduced, as presented in Fig 5A. The shaded areas between days 1 to 5 and days 7 to 13 represent the AUC in the period of antibiotic administration (Table H in S1 File) and the recovery period of post administration (Table I in S1 File), respectively.

As displayed in Fig 5B, during antibiotic administration, the AUC of fecal SCFAs, especially the high abundant SCFAs, such as acetic acid, propionic acid and butyric acid, were significantly reduced (*p* <0.05), except for isobutyric acid and hexanoic acid, in the antibiotic-treated groups compared with the control group. Effects of cefdinir on SCFAs were more pronounced than those of azithromycin. During post administration period, the AUC values for acetic acid, propionic acid, butyric acid and valeric acid in the cefdinir group were remarkably reduced (*p* <0.01) compared with those in the control group, but were higher than those during the period of drug administration. During the recovery period, the AUC of isobutyric acid was significantly increased (*p* <0.01) and the AUC of isovaleric acid showed no significant difference (*p* >0.05) compared with the control group, indicating that the bacteria which produced isobutyric acid and isovaleric acid, the branched-chain fatty acids, rapidly returned to the control level. The AUC of acetic acid, propionic acid, isobutyric acid, butyric acid, isovaleric acid and valeric acid in the azithromycin-treated group were obviously decreased (*p* <0.05) compared with the control group and none of them recovered to the control level within 8 days, except for hexanoic acid. These disparate results could be partially due to different antimicrobial spectrum and activities of cefdinir and azithromycin [46,47].

### Overall quantities and relative proportions of seven SCFAs in fecal samples

**Fig 6:**
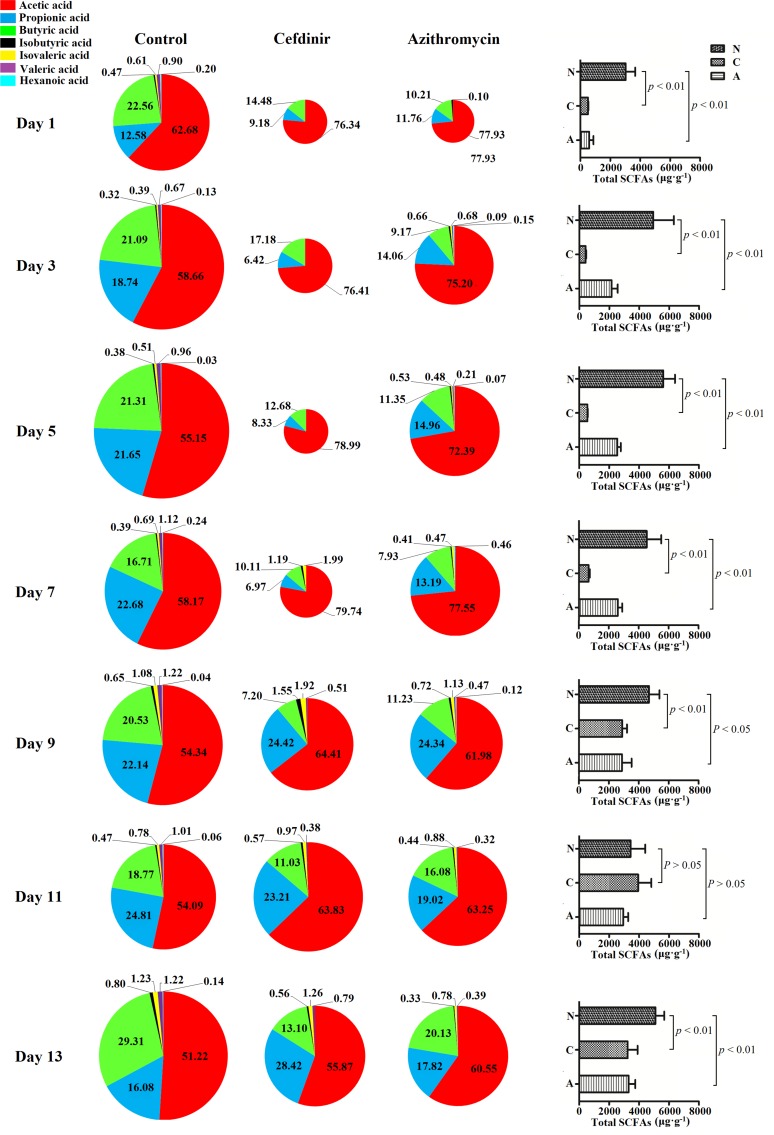
Relative proportions of seven SCFAs in fecal samples from the control, cefdinir-treated or azithromycin-treated groups during and post-antibiotic administration. (The size of the pie charts represents the relative total quantity of SCFAs; “N” represents the control group. “C” represents the cefdinir group. “A” represents the azithromycin group).

As illustrated in Fig 6, compared with the control group, the overall quantities and relative proportions of SCFAs fluctuated remarkably throughout the study in the antibiotic-treated groups. Quantities and proportions of SCFAs produced by the gut microbiota remained relative stable in the control rats. During days 1 and 13, the mean quantities of total SCFAs in the control group ranged from 3026.6 μg·g^-1^ to 5616.4 μg·g^-1^ with the distribution of proportion: acetic acid 56.33% ± 3.78% (mean ± SD), propionic acid 19.81% ± 4.27%, butyric acid 21.47% ± 3.95%, isobutyric acid 0.50% ± 0.17%, isovaleric acid 0.76% ± 0.30%, valeric acid 1.02% ± 0.19%, and hexanoic acid 0.12% ± 0.08%, respectively.

During cefdinir treatment (days 1, 3 and 5), the quantities and proportions of the seven SCFAs in fecal samples were changed markedly compared with the control group. In detail, compared with the control group, the total SCFAs decreased significantly (*p* <0.01) and mostly were undetectable except acetic acid, propionic acid and butyric acid. The ratio of these three remaining SCFAs was also dramatically altered, as follows: acetic acid 77.25% ± 1.51%, propionic acid 7.97% ± 1.41%, and butyric acid 14.78% ± 2.26%. In the recovery period following cefdinir administration (days 7, 9, 11 and 13), the mean quantities of total SCFAs were restored from 642.9 μg·g^-1^ to 3942.8 μg·g^-1^, but it did not reach the normal level of the control group (*p* <0.01) except at day 11. The ratio of the SCFAs gradually returned to control levels eventually with acetic acid at 55.87% ± 8.65%, propionic acid at 28.42% ± 6.61%, butyric acid at 13.10% ± 7.52%, isobutyric acid at 0.56% ± 0.18%, isovaleric acid at 1.26% ± 0.35%, and valeric acid at 0.79% ± 0.66%, respectively. In azithromycin-treated group, the overall quantities and relative proportions of the SCFAs dramatically changed within 24 h after the first administration. In day 3 and 5, the quantities and proportions of SCFAs recovered compared with day 1. The mean quantities of total SCFAs in the azithromycin group were higher than that in the cefdinir group during administration period with a concentration between 590.5 μg·g^-1^ and 2552.3 μg·g^-1^, which reduced significantly (*p* <0.01) compared with the control group. During post-administration recovery (days 7, 9, 11 and 13), the mean quantities of total SCFAs increased gradually from 2600.0 μg·g^-1^ to 3308.8 μg·g^-1^, however, did not recover to normal level (*p* <0.05) except at day 11. The ratios of the seven SCFAs in the azithromycin group gradually returned to the ratios in the control group with 60.55% ± 7.47% for acetic acid, 17.82% ± 3.34% for propionic acid, 20.13% ± 6.91% for butyric acid, 0.33% ± 0.06% for isobutyric acid, 0.78% ± 0.28% for isovaleric acid, 0.39% ± 0.07% for valeric acid at day 13, respectively.

Early studies suggested that SCFAs produced in the intestinal tract might inhibit the survival of harmful or conditional pathogenic bacteria [13], leading to protection of the intestinal tract against infection and inflammation [12]. Loss of total bacteria, especially loss of beneficial bacteria in the gut may result in decreases in diversity and quantities of SCFAs in the feces of antibiotic-treated rats.

Conventional studies have shown that antibiotics can disrupt the equilibrium of gut microbial communities [22–25]. Traditionally, disruption of the richness and diversity indices of gut microbial communities are often used to interpret the side effects of oral antibiotics [48], such disruption is one of the probable mechanisms how antibiotics influence gut SCFAs.

Our new method has potential clinical applications if SCFAs can serve as a personalized drug safety and efficacy index. Antibiotic treatments should be guided according to the profile of SCFAs.

## Conclusions

A rapid and quantitative GC method was developed for the analysis of fecal SCFAs of rats and applied to investigate the influences of cefdinir and azithromycin in a time–saving manner. Using this new method, we unveiled that a short course of antibiotic treatment resulted in a significant reduction in the sum, diversity and structure of the SCFAs in fecal samples. The fluctuation range and proportional change of SCFAs could provide more insights to evaluate the effects of new antibiotics in future clinical trials

## Supporting Information

S1 File Tables A-I. The concentrations (μg·g^-1^) of seven short-chain fatty acids in antibiotic-treated and recovery period (Tables A-G). Area under concentration *versus* time curve (AUC) of fecal SCFAs in fecal samples (Tables H-I).
(DOC)
