# Supplementary material for: Sensitive and Simplified Detection of Antibiotic Influence on the Dynamic and Versatile Changes of Fecal Short-Chain Fatty Acids
Source: PLoS One. 2016 Dec 1;11(12):e0167032. doi: 10.1371/journal.pone.0167032 (PMC5132400; doi:10.1371/journal.pone.0167032)
Supplement: S1 File — Tables A-I. The concentrations (μg·g-1) of seven short-chain fatty acids in antibiotic-treated and recovery period (Tables A-G). Area under concentration versus time curve (AUC) of fecal SCFAs in fecal samples (Tables H-I). (DOC) [file pone.0167032.s001.doc]

**Supporting Information to:**

Sensitive and simplified detection of antibiotic influence on the dynamic and versatile changes of fecal short-chain fatty acids

Xiaoya Zhao1,2¶, Zhenzuo Jiang1,2¶, Fan Yang1,2, Yan Wang1,2, Xiumei Gao1, Yuefei Wang1,2*, Xin Chai1,2, Guixiang Pan1,2, Yan Zhu1,2*

|  |
| --- |

^1^ Tianjin State Key Laboratory of Modern Chinese Medicine, Tianjin University of Traditional Chinese Medicine, Tianjin 300193, China

^2^ Research and Development Center of TCM, Tianjin International Joint Academy of Biotechnology and Medicine, Tianjin 300457, China

* Corresponding author

E-mail: [wangyuefei_2006@hotmail.com](mailto:wangyuefei_2006@hotmail.com) (YFW); [yanzhu.harvard@icloud.com](mailto:yanzhu.harvard@icloud.com)

**^¶^** These authors contributed equally to this work.

**Table A** The concentration of acetic acid in administration and recovery period (μg·g^-1^)

| *No. | Days | | | | | | |
| --- | --- | --- | --- | --- | --- | --- | --- |
|  | 1 | 3 | 5 | 7 | 9 | 11 | 13 |
| N1 | 2424.8 | 3339.7 | 2744.5 | 2242.0 | 2709.8 | 2439.0 | 2783.2 |
| N2 | 2129.3 | 2327.9 | 3077.0 | 3013.9 | 2860.7 | 1777.1 | 2745.8 |
| N3 | 1854.1 | 2194.0 | 3224.4 | 3225.1 | 2077.0 | 1579.4 | 2764.1 |
| N4 | 1354.8 | 2891.2 | 3183.5 | 3080.8 | 3004.2 | 2403.1 | 2680.2 |
| N5 | 1937.7 | 3810.8 | 3319.0 | 1656.1 | 2518.4 | 1482.2 | 2195.9 |
| N6 | 1597.1 | 2540.8 | 2875.4 | 2384.3 | 2059.4 | 1358.5 | 2449.5 |
| $\bar{x}$ | 1883.0 | 2850.7 | 3070.6 | 2600.4 | 2538.3 | 1839.9 | 2603.1 |
| SD | 379.18 | 626.61 | 220.27 | 609.75 | 398.23 | 470.66 | 234.13 |
| C1 | 349.50 | 299.95 | 413.35 | 525.33 | 2071.2 | 2120.2 | 1706.0 |
| C2 | 390.43 | 326.49 | 437.97 | 416.99 | 1869.3 | 1842.6 | 1245.5 |
| C3 | 393.22 | 287.99 | 401.96 | 616.24 | 1485.5 | 2468.0 | 1829.4 |
| C4 | 376.56 | 320.89 | 449.09 | 482.38 | 2108.5 | 2888.5 | 2506.3 |
| C5 | 403.23 | 324.23 | 458.89 | 541.35 | 1854.5 | 2773.3 | 1987.5 |
| C6 | 434.67 | 340.07 | 464.99 | 567.91 | 1786.1 | 2792.2 | 1540.1 |
| $\bar{x}$ | 391.27 | 316.60 | 437.71 | 525.03 | 1862.5 | 2480.8 | 1802.5 |
| SD | 28.257 | 19.084 | 25.273 | 69.158 | 224.58 | 421.02 | 428.67 |
| A1 | 282.66 | 1217.3 | 1707.6 | 1710.9 | 2094.1 | 2005.1 | 2147.3 |
| A2 | 324.46 | 1273.4 | 1650.3 | 1891.4 | 1545.7 | 2070.5 | 1904.6 |
| A3 | 363.16 | 1375.5 | 2047.6 | 1804.3 | 2196.1 | 1986.1 | 1875.8 |
| A4 | 874.48 | 1542.7 | 1550.1 | 1691.8 | 1391.3 | 1834.4 | 2011.9 |
| A5 | 489.49 | 2159.4 | 2118.5 | 2467.2 | 1565.3 | 1612.6 | 1877.7 |
| A6 | 418.47 | 2316.7 | 2038.9 | 1947.6 | 1834.5 | 1642.3 | 2058.2 |
| $\bar{x}$ | 458.79 | 1647.5 | 1852.2 | 1918.9 | 1771.2 | 1858.5 | 1979.2 |
| SD | 216.14 | 473.26 | 243.67 | 286.41 | 324.49 | 195.20 | 111.46 |

*: “N” represents the control group. “C” represents the cefdinir group. “A” represents the azithromycin group.

**Table B** The concentration of propionic acid in administration and recovery period (μg·g^-1^)

| *No. | Days | | | | | | |
| --- | --- | --- | --- | --- | --- | --- | --- |
|  | 1 | 3 | 5 | 7 | 9 | 11 | 13 |
| N1 | 440.8 | 1050 | 1505 | 1214 | 865.3 | 936.2 | 1012 |
| N2 | 257.4 | 538.7 | 1035 | 1478 | 1960 | 1254 | 1137 |
| N3 | 441.8 | 254.9 | 1142 | 1143 | 1002 | 1110 | 754.3 |
| N4 | 430.1 | 1512 | 1237 | 1325 | 1156 | 826.0 | 554.7 |
| N5 | 216.6 | 1133 | 1041 | 609.4 | 709.4 | 465.1 | 628.3 |
| N6 | 370.6 | 1206 | 1167 | 929.8 | 583.6 | 483.0 | 852.5 |
| $\bar{x}$ | 359.6 | 949.1 | 1188 | 1117 | 1046 | 845.8 | 823.2 |
| SD | 99.33 | 464.2 | 173.4 | 308.7 | 491.9 | 323.1 | 223.6 |
| C1 | 44.68 | – | 47.82 | 66.69 | 730.8 | 792.1 | 574.3 |
| C2 | 47.17 | 41.59 | 43.96 | 41.23 | 658.7 | 661.1 | 835.8 |
| C3 | 47.32 | – | 49.22 | 81.16 | 584.9 | 935.4 | 648.4 |
| C4 | 47.61 | 42.36 | 43.48 | 46.20 | 867.3 | 1402 | 1182 |
| C5 | 44.83 | 43.02 | 46.29 | 46.89 | 660.3 | 533.7 | 1019 |
| C6 | 49.98 | 42.74 | 45.31 | 49.63 | 732.6 | 1233 | 1273 |
| $\bar{x}$ | 46.93 | 42.43 | 46.01 | 55.30 | 705.8 | 926.3 | 922.1 |
| SD | 1.972 | 0.6190 | 2.225 | 15.37 | 96.27 | 335.6 | 284.0 |
| A1 | 48.21 | 377.9 | 379.6 | 374.4 | 913.4 | 416.5 | 370.9 |
| A2 | 47.84 | 334.0 | 432.6 | 451.8 | 481.3 | 642.5 | 585.0 |
| A3 | 52.90 | 249.9 | 339.1 | 398.3 | 965.9 | 643.1 | 655.3 |
| A4 | 173.0 | 371.2 | 383.4 | 421.0 | 634.6 | 637.2 | 776.8 |
| A5 | 46.05 | 194.3 | 396.5 | 422.9 | 581.3 | 482.0 | 593.8 |
| A6 | 67.69 | 192.3 | 332.9 | 331.2 | 656.2 | 544.7 | 557.3 |
| $\bar{x}$ | 72.61 | 286.6 | 377.4 | 399.9 | 705.5 | 561.0 | 589.9 |
| SD | 49.79 | 85.44 | 37.14 | 42.54 | 191.9 | 96.52 | 132.9 |

*: “N” represents the control group. “C” represents the cefdinir group. “A” represents

the azithromycin group.

“–” represents the measured values below the detection limit.

**Table C** The concentration of isobutyric acid in administration and recovery period (μg·g^-1^)

| *No. | Days | | | | | | |
| --- | --- | --- | --- | --- | --- | --- | --- |
|  | 1 | 3 | 5 | 7 | 9 | 11 | 13 |
| N1 | 13.4 | 22.6 | 13.8 | 14.3 | 15.4 | 21.5 | 43.0 |
| N2 | 8.68 | 16.3 | 17.1 | 15.1 | 17.9 | 12.0 | 58.3 |
| N3 | 9.01 | 10.8 | 21.5 | 21.5 | 20.5 | 12.4 | 41.8 |
| N4 | 13.1 | 8.60 | 17.1 | 12.0 | 27.9 | 21.6 | 35.2 |
| N5 | 23.7 | 25.2 | 43.1 | 16.5 | 68.7 | 8.88 | 36.3 |
| N6 | 15.2 | 11.6 | 19.8 | 15.8 | 31.4 | 19.2 | 31.4 |
| $\bar{x}$ | 13.8 | 15.9 | 22.0 | 15.9 | 30.3 | 15.9 | 41.0 |
| SD | 5.46 | 6.75 | 10.6 | 3.16 | 19.8 | 5.51 | 9.48 |
| C1 | – | – | – | – | 54.2 | 21.9 | 25.4 |
| C2 | – | – | – | – | 42.6 | 12.8 | 8.73 |
| C3 | – | – | – | – | 41.4 | 20.4 | 15.6 |
| C4 | – | – | – | – | 42.4 | 32.0 | 24.7 |
| C5 | – | – | – | – | 47.2 | 15.2 | 11.5 |
| C6 | – | – | – | 8.44 | 39.2 | 37.0 | 24.4 |
| $\bar{x}$ | – | – | – | 8.44 | 44.5 | 23.2 | 18.4 |
| SD | – | – | – | – | 5.43 | 9.48 | 7.39 |
| A1 | – | 12.7 | 15.9 | 11.3 | 25.5 | 7.25 | 8.65 |
| A2 | – | 11.6 | 15.0 | 15.1 | 15.8 | 10.7 | 8.56 |
| A3 | – | 13.9 | 11.3 | 11.0 | 24.6 | 12.7 | 13.2 |
| A4 | 7.30 | 11.7 | 17.2 | 11.4 | 23.5 | 27.0 | 13.9 |
| A5 | – | 17.6 | 7.76 | 9.02 | 14.0 | 7.70 | 9.88 |
| A6 | – | 17.2 | 12.0 | 10.2 | 20.1 | 13.9 | 10.3 |
| $\bar{x}$ | 7.30 | 14.1 | 13.2 | 11.3 | 20.6 | 13.2 | 10.8 |
| SD | – | 2.70 | 3.50 | 2.03 | 4.80 | 7.26 | 2.30 |

*: “N” represents the control group. “C” represents the cefdinir group. “A” represents the azithromycin group.

“–” represents the measured values below the detection limit.

**Table** **D** The concentration of butyric acid in administration and recovery period (μg·g^-1^)

| *No. | Days | | | | | | |
| --- | --- | --- | --- | --- | --- | --- | --- |
|  | 1 | 3 | 5 | 7 | 9 | 11 | 13 |
| N1 | 711.0 | 1305 | 645.2 | 600.4 | 660.1 | 993.4 | 1225 |
| N2 | 708.2 | 1073 | 1421 | 768.5 | 413.8 | 402.3 | 1532 |
| N3 | 857.1 | 724.4 | 812.4 | 812.6 | 513.8 | 405.0 | 2080 |
| N4 | 416.5 | 723.0 | 987.8 | 896.0 | 1306 | 1348 | 1511 |
| N5 | 1369 | 1976 | 2562 | 612.4 | 1203 | 390.9 | 1185 |
| N6 | 241.3 | 559.9 | 1059 | 685.0 | 1669 | 510.3 | 1467 |
| $\bar{x}$ | 717.2 | 1060 | 1248 | 729.1 | 960.9 | 674.9 | 1500 |
| SD | 390.3 | 524.5 | 694.6 | 117.1 | 503.8 | 402.2 | 320.4 |
| C1 | 74.44 | 71.76 | 68.71 | – | 221.8 | 439.9 | 640.6 |
| C2 | 75.40 | 71.08 | 70.08 | 68.43 | 226.5 | 187.7 | 173.9 |
| C3 | 75.08 | 70.43 | 70.98 | 68.97 | 174.8 | 521.6 | 367.8 |
| C4 | 73.54 | 69.20 | 71.56 | 71.58 | 195.0 | 276.1 | 299.9 |
| C5 | 72.48 | 70.39 | 69.04 | 71.73 | 235.8 | 288.6 | 207.9 |
| C6 | 72.63 | 71.96 | 70.39 | 71.98 | 187.7 | 1014 | 918.8 |
| $\bar{x}$ | 73.93 | 70.80 | 70.13 | 70.54 | 207.0 | 454.6 | 434.8 |
| SD | 1.240 | 1.022 | 1.097 | 1.695 | 24.42 | 299.3 | 289.4 |
| A1 | 71.98 | 214.1 | 416.1 | 313.4 | 476.0 | 413.0 | 495.3 |
| A2 | 70.96 | 196.2 | 184.6 | 266.8 | 222.0 | 639.2 | 896.0 |
| A3 | 87.69 | 215.4 | 388.7 | 292.2 | 486.8 | 337.5 | 446.7 |
| A4 | 116.7 | 160.7 | 266.6 | 191.4 | 237.6 | 629.9 | 1257 |
| A5 | – | 176.1 | 225.3 | 244.4 | 249.5 | 350.0 | 380.2 |
| A6 | – | 183.1 | 261.8 | 199.1 | 317.0 | 496.6 | 664.6 |
| $\bar{x}$ | 86.84 | 191.0 | 290.5 | 251.2 | 331.5 | 477.7 | 689.9 |
| SD | 21.34 | 21.73 | 91.93 | 49.27 | 120.6 | 134.0 | 333.9 |

*: “N” represents the control group. “C” represents the cefdinir group. “A” represents the azithromycin group.

“–” represents the measured values below the detection limit.

**Table E** The concentration of isovaleric acid in administration and recovery period (μg·g^-1^)

| *No. | Days | | | | | | |
| --- | --- | --- | --- | --- | --- | --- | --- |
|  | 1 | 3 | 5 | 7 | 9 | 11 | 13 |
| N1 | 17.4 | 35.2 | 22.8 | 22.0 | 21.0 | 36.9 | 68.7 |
| N2 | 11.4 | 15.6 | 19.6 | 20.0 | 25.9 | 15.3 | 91.0 |
| N3 | 9.23 | 16.2 | 26.9 | 26.9 | 34.6 | 25.2 | 63.0 |
| N4 | 16.4 | 10.6 | 18.0 | 17.3 | 37.9 | 32.6 | 50.7 |
| N5 | 29.9 | 26.5 | 64.0 | 32.6 | 130 | 11.9 | 55.7 |
| N6 | 22.0 | 11.1 | 27.3 | 28.2 | 51.3 | 35.5 | 48.5 |
| $\bar{x}$ | 17.7 | 19.2 | 29.8 | 24.5 | 50.2 | 26.2 | 63.0 |
| SD | 7.47 | 9.72 | 17.2 | 5.71 | 40.6 | 10.6 | 15.7 |
| C1 | – | – | – | – | 61.2 | 34.8 | 48.6 |
| C2 | – | – | – | – | 52.4 | 24.3 | 22.5 |
| C3 | – | – | – | – | 50.5 | 36.9 | 37.1 |
| C4 | – | – | – | – | 54.8 | 50.8 | 48.9 |
| C5 | – | – | – | – | 67.2 | 24.1 | 26.2 |
| C6 | – | – | – | 14.2 | 44.1 | 65.4 | 66.5 |
| $\bar{x}$ | – | – | – | 14.2 | 55.0 | 39.4 | 41.6 |
| SD | – | – | – | – | 8.16 | 16.1 | 16.4 |
| A1 | – | 17.7 | 11.5 | 12.6 | 43.8 | 11.7 | 17.3 |
| A2 | – | 12.0 | 16.2 | 16.8 | 17.6 | 21.2 | 18.6 |
| A3 | – | 11.2 | 8.25 | 14.5 | 42.9 | 37.8 | 39.8 |
| A4 | – | 11.1 | 16.1 | 14.0 | 41.6 | 48.9 | 35.2 |
| A5 | – | 17.0 | 8.37 | 9.48 | 19.3 | 10.5 | 22.3 |
| A6 | – | 18.0 | 11.7 | 11.7 | 30.7 | 28.7 | 21.6 |
| $\bar{x}$ | – | 14.5 | 12.0 | 13.2 | 32.7 | 26.5 | 25.8 |
| SD | – | 3.39 | 3.52 | 2.51 | 12.0 | 15.1 | 9.36 |

*: “N” represents the control group. “C” represents the cefdinir group. “A” represents the azithromycin group.

“–” represents the measured values below the detection limit.

**Table F** The concentration of valeric acid in administration and recovery period (μg·g^-1^)

| *NO. | Days | | | | | | |
| --- | --- | --- | --- | --- | --- | --- | --- |
|  | 1 | 3 | 5 | 7 | 9 | 11 | 13 |
| N1 | 34.61 | 53.79 | 38.62 | 35.72 | 28.13 | 44.91 | 53.46 |
| N2 | – | 13.90 | 55.99 | 48.46 | 31.80 | 19.37 | 69.53 |
| N3 | 40.57 | 14.20 | 43.39 | 43.40 | 49.75 | 33.59 | 69.26 |
| N4 | 23.48 | 25.18 | 36.68 | 33.02 | 49.59 | 50.11 | 64.52 |
| N5 | 58.30 | 89.07 | 109.6 | 43.82 | 120.9 | 23.79 | 54.42 |
| N6 | 12.83 | 24.80 | 51.45 | 45.76 | 56.94 | 34.20 | 60.51 |
| $\bar{x}$ | 33.96 | 36.82 | 55.96 | 41.70 | 56.18 | 34.33 | 61.95 |
| SD | 17.27 | 29.44 | 27.33 | 6.011 | 33.61 | 11.80 | 7.048 |
| C1 | – | – | – | – | 15.50 | 15.44 | 45.15 |
| C2 | – | – | – | – | 14.31 | 10.70 | 11.03 |
| C3 | – | – | – | – | 14.21 | 13.25 | 13.45 |
| C4 | – | – | – | – | 12.81 | 11.12 | 10.66 |
| C5 | – | – | – | – | 18.31 | 10.40 | 9.62 |
| C6 | – | – | – | – | 11.97 | 31.28 | 69.11 |
| $\bar{x}$ | – | – | – | – | 14.52 | 15.37 | 26.50 |
| SD | – | – | – | – | 2.231 | 8.028 | 24.94 |
| A1 | – | 9.937 | 11.57 | – | 17.16 | 9.280 | 11.56 |
| A2 | – | – | – | 9.664 | 10.60 | 10.62 | 11.11 |
| A3 | – | – | 11.01 | – | 16.78 | 13.42 | 13.58 |
| A4 | – | – | 9.786 | – | 12.43 | 13.68 | 21.06 |
| A5 | – | – | – | – | 11.49 | – | 10.74 |
| A6 | – | – | – | – | 12.17 | 11.31 | 11.14 |
| $\bar{x}$ | – | 9.937 | 10.79 | 9.664 | 13.44 | 11.66 | 13.20 |
| SD | – | – | 0.9106 | – | 2.812 | 1.876 | 3.981 |

*: “N” represents the control group. “C” represents the cefdinir group. “A” represents the azithromycin group.

“–” represents the measured values below the detection limit.

**Table G** The concentration of hexanoic acid in administration and recovery period (μg·g^-1^)

| *NO. | Days | | | | | | |
| --- | --- | --- | --- | --- | --- | --- | --- |
|  | 1 | 3 | 5 | 7 | 9 | 11 | 13 |
| N1 | – | – | – | – | – | – | – |
| N2 | 9.353 | 11.64 | – | – | – | – | 8.917 |
| N3 | 9.383 | 9.717 | – | – | – | 11.26 | 10.62 |
| N4 | – | – | – | 9.657 | – | – | 11.31 |
| N5 | 23.27 | 12.77 | 12.49 | 11.03 | 10.70 | – | 11.66 |
| N6 | – | – | – | 10.02 | – | – | – |
| $\bar{x}$ | 14.00 | 11.37 | 12.49 | 10.23 | 10.70 | 11.26 | 10.63 |
| SD | 8.027 | 1.542 | – | 0.710 | – | – | 1.220 |
| C1 | – | – | – | – | – | – | – |
| C2 | – | – | – | – | – | – | – |
| C3 | – | – | – | – | – | 8.826 | – |
| C4 | – | – | – | – | – | – | – |
| C5 | – | – | – | – | – | – | – |
| C6 | – | – | – | – | – | 10.42 | – |
| $\bar{x}$ | – | – | – | – | – | 9.623 | – |
| SD | – | – | – | – | – | – | – |
| A1 | – | – | – | – | – | – | – |
| A2 | – | – | – | 11.42 | – | – | – |
| A3 | – | – | – | – | 10.60 | – | – |
| A4 | – | – | 9.993 | – | – | – | – |
| A5 | – | 10.34 | – | – | – | – | – |
| A6 | – | 13.11 | – | 11.52 | 13.39 | – | – |
| $\bar{x}$ | – | 11.72 | 9.993 | 11.47 | 12.00 | – | – |
| SD | – | – | – | – | – | – | – |

*: “N” represents the control group. “C” represents the cefdinir group. “A” represents the azithromycin group.

“–” represents the measured values below the detection limit.

**Table H** Area under concentration *versus* time curve (AUC) of fecal SCFA in fecal samples in the period of antibiotic administration (μg·g^-1^·d)

| *NO | AA | PA | *i*-BA | BA | *i*-VA | VA | HA |
| --- | --- | --- | --- | --- | --- | --- | --- |
|
| N1 | 13061.09 | 4266.75 | 79.17 | 4321.13 | 119.29 | 198.10 | – |
| N2 | 10926.83 | 2498.86 | 62.73 | 4628.47 | 67.86 | 90.74 | 25.67 |
| N3 | 10393.50 | 2314.97 | 56.69 | 3546.91 | 73.16 | 132.64 | 23.79 |
| N4 | 10997.96 | 4906.23 | 53.91 | 3058.53 | 63.74 | 122.27 | – |
| N5 | 13847.30 | 3630.99 | 128.86 | 8568.03 | 161.93 | 375.23 | 72.92 |
| N6 | 10352.71 | 4135.43 | 65.93 | 2540.96 | 82.47 | 120.30 | – |
| C1 | 1537.49 | 207.34 | – | 323.89 | – | – | – |
| C2 | 1676.58 | 197.90 | – | 325.33 | – | – | – |
| C3 | 1567.78 | 216.73 | – | 324.45 | – | – | – |
| C4 | 1655.71 | 199.61 | – | 320.27 | – | – | – |
| C5 | 1712.20 | 199.58 | – | 318.53 | – | – | – |
| C6 | 1797.14 | 205.76 | – | 323.25 | – | – | – |
| A1 | 4566.25 | 1207.66 | 47.68 | 952.34 | 55.75 | 36.41 | – |
| A2 | 4683.70 | 1172.31 | 43.94 | 683.49 | 46.12 | – | – |
| A3 | 5343.33 | 918.18 | 46.02 | 951.08 | 36.19 | 27.52 | – |
| A4 | 5947.27 | 1385.28 | 51.47 | 763.15 | 43.96 | 24.46 | 24.98 |
| A5 | 7171.58 | 854.26 | 51.85 | 665.71 | 50.95 | – | 15.51 |
| A6 | 7300.03 | 819.05 | 55.03 | 719.40 | 56.62 | – | 19.66 |

*: “N” represents the control group. “C” represents the cefdinir group. “A” represents the azithromycin group.

“–” represents the measured values below the detection limit.

AA: acetic acid; PA: propionic acid; *i*-BA: isobutyric acid; BA:butyric acid; *i*-VA: isovaleric acid; VA: valeric acid; HA: hexanoic acid

**Table I** Area under concentration *versus* time curve (AUC) of fecal SCFA in fecal samples in the recovery period of post-administration (μg·g^-1^·d)

| *NO | AA | PA | *i*-BA | BA | *i*-VA | VA | HA |
| --- | --- | --- | --- | --- | --- | --- | --- |
|
| N1 | 23169.57 | 10077.27 | 181.02 | 7233.82 | 283.34 | 360.29 | – |
| N2 | 25584.20 | 14217.95 | 185.68 | 6621.89 | 263.64 | 389.98 | 57.96 |
| N3 | 24589.86 | 10121.30 | 204.40 | 7574.73 | 303.94 | 431.24 | 83.82 |
| N4 | 27358.43 | 10482.45 | 188.31 | 10849.83 | 269.81 | 412.52 | 96.71 |
| N5 | 17649.49 | 5719.85 | 265.49 | 7127.93 | 487.01 | 540.91 | 105.04 |
| N6 | 20014.95 | 7170.02 | 203.79 | 8908.85 | 348.87 | 448.72 | 35.06 |
| C1 | 12452.87 | 3920.13 | 367.59 | 2740.47 | 454.61 | 161.27 | – |
| C2 | 10545.85 | 3660.96 | 268.93 | 1310.32 | 359.29 | 111.13 | – |
| C3 | 12509.50 | 4054.29 | 284.17 | 2071.00 | 388.61 | 118.13 | 48.54 |
| C4 | 14671.14 | 5929.82 | 321.72 | 1564.23 | 451.78 | 103.37 | – |
| C5 | 13679.17 | 3618.23 | 301.28 | 1579.58 | 444.42 | 131.13 | – |
| C6 | 13252.24 | 5426.77 | 214.80 | 3645.41 | 349.44 | 197.52 | 57.31 |
| A1 | 18044.78 | 4715.51 | 124.84 | 3683.47 | 184.85 | 124.52 | – |
| A2 | 17648.48 | 4865.74 | 129.17 | 3818.75 | 171.61 | 97.03 | 39.98 |
| A3 | 18359.61 | 5665.59 | 137.40 | 3410.31 | 266.57 | 132.71 | 47.69 |
| A4 | 16076.41 | 5215.17 | 166.20 | 3852.81 | 279.51 | 116.77 | – |
| A5 | 19335.81 | 4623.73 | 93.89 | 2678.84 | 124.57 | 96.18 | – |
| A6 | 17775.88 | 4449.47 | 124.25 | 3187.61 | 193.08 | 100.69 | 65.25 |

*: “N” represents the control group. “C” represents the cefdinir group. “A” represents the azithromycin group.

“–” represents the measured values below the detection limit.

AA: acetic acid; PA: propionic acid; *i*-BA: isobutyric acid; BA:butyric acid; *i*-VA: isovaleric acid; VA: valeric acid; HA: hexanoic acid
